# A new species of *Symplocos* (Symplocaceae) from southern Ecuador

**DOI:** 10.3897/phytokeys.56.5306

**Published:** 2015-09-24

**Authors:** Carmen Ulloa Ulloa, Bertil Ståhl, Danilo Minga, Raffaella Ansaloni

**Affiliations:** 1 Investigador Prometeo, Facultad de Ciencia y Tecnología, Universidad del Azuay, Cuenca, Ecuador; 2 Missouri Botanical Garden, P.O. Box 299, St. Louis, MO 63166, U.S.A.; 3 Department of Organismal Biology, Uppsala University Campus Gotland, SE62167 Visby, Sweden; 4 Facultad de Ciencia y Tecnología, Universidad del Azuay, Av. 24 de Mayo 7-77, Cuenca, Ecuador

**Keywords:** *Symplocos*, Ecuador, Andes

## Abstract

A new species from Ecuador, *Symplocos limonensis*, is here described and illustrated. It resembles *Symplocos clethrifolia* but differs by having larger leaves with evident (i.e., not concealed) areoles on lower surface, sessile inflorescences, smaller white corollas, and fewer stamens. The species is only known from three collections in the Andean forests of Morona-Santiago Province in southern Ecuador.

## Abstract (es)

Se describe e ilustra una nueva especie, *Symplocos limonensis*, de Ecuador. Se parece a *Symplocos clethrifolia* pero se diferencia por tener las hojas más grandes, con las aréolas evidentes (no ocultas) en el envés, las inflorescencias sésiles y las flores más pequeñas, blancas y con menos estambres. La especie solo se conoce de tres ejemplares de los bosques andinos de la provincia de Morona-Santiago en el sur del país.

## Introduction

The genus *Symplocos* Jacq. is represented in Ecuador by ca. 33 species (Ståhl 2010a), the majority of which occur in Andean forests at 2500–3500 m elevation (Ståhl 1995). Thirteen of the species are country endemics (Barriga 2011) and have been described in the last 25 years, significantly increasing the knowledge of this still poorly known genus. In his treatment of the Symplocaceae for the Flora of Ecuador series, Ståhl (1991) described several new species of *Symplocos*, especially from southern Ecuador. One of these, *Symplocos clethrifolia* Ståhl, was based on five collections, one of which (*Ulloa 487*) was indicated to deviate from the remaining four in floral and leaf features. This collection was provisionally treated under *Symplocos clethrifolia* until more material could help elucidate its taxonomic status.

## Materials and methods

Recent examination of the *Symplocos* collections in Ecuadorian herbaria revealed additional gatherings from the same general area that have led us to conclude that the Ulloa collection, along with some new ones, belong to a previously undescribed species, which is described here. Acronyms of the herbaria follow Thiers (2015).

## Description of the new species

### Symplocos limonensis

Taxon classificationPlantaeEricalesSymplocaceae

Ståhl, C. Ulloa & Minga sp. nov.

urn:lsid:ipni.org:names:77150152-1

Fig. 1

#### Diagnosis.

*Symplocos limonensis* differs from *Symplocos clethrifolia* Ståhl by having larger leaves (up to 13.5 vs. 9.5 cm) with evident (i.e., not concealed) venation areoles abaxially (vs. densely ferrugineous-velutinous with matted hairs concealing areoles), sessile inflorescences (vs. pedunculate), somewhat smaller, white (vs. red to pink) corollas, and fewer stamens (40–50 vs. 60–70).

Type: ECUADOR. Morona-Santiago: road Cuenca-Macas (road Gualaceo-Limón East of the pass), 2700–3400 m, 19 Aug 1987, *C. Ulloa 487* (Holotype: QCA-209874!; isotypes AAU!, GB!, MO-6500819!).

Tree or treelet to 12 m tall; branchlets reddish brown, sparsely villous to glabrescent, apical buds densely long-villous. Leaves alternate, petiolate; blade oblong to widely elliptic, 8–13.5 × 4–9 cm, the blades exceptionally almost circular and ca. 3 × 3 cm, coriaceous, light green and long-villous beneath, olive green and glabrous above, base rounded or truncate, slightly decurrent on petiole, apex rounded to widely obtuse, mucronulate at the tip, margins denticulate, teeth 3–4 mm apart, lateral veins 8–10 per side, midvein, lateral veins, and veinlets on lower side prominently raised and verrucose, impressed above; petiole 1–1.5 cm long, rounded and long-villous beneath, flattened and glabrous above. Inflorescences fasciculate, sessile, borne in axils of extant leaves and along branchlets beneath the foliage; flowers 8–10 per inflorescence; bracts 4, very broadly ovate, 2–3 × 2–2.5 mm, strigulose on midvein toward apex, otherwise glabrous, margins ciliolate. Flowers with the calyx synsepalous, tube ca. 1.5 mm long, lobes 5, very broadly ovate, 1.5–2.5 × 2.2– 2.8 mm, margins ciliolate; corolla sympetalous, glabrous, white, 5–6 mm long, lobes 5 to 7, broadly oblong, 1.8–4 mm wide, margins entire; stamens 40–45 in 3(4) rows, filaments smooth, fused for ca. 2 mm to the corolla tube, those of the inner whorl fused most of their length leaving ca. 0.5 mm of the filaments free, stamens of the outer whorls with free filaments 1–2.8 mm long, more or less flattened and constricted at apex, anthers ca. 0.5 mm long; disc intrastaminal, dome-shaped, densely villous; style ca. 2.5 mm long, glabrous; stigma capitate and irregularly 3-lobed; ovary inferior, 3-locular with 3 or 4 ovules per locule. Mature fruits not seen.

**Figure 1.**
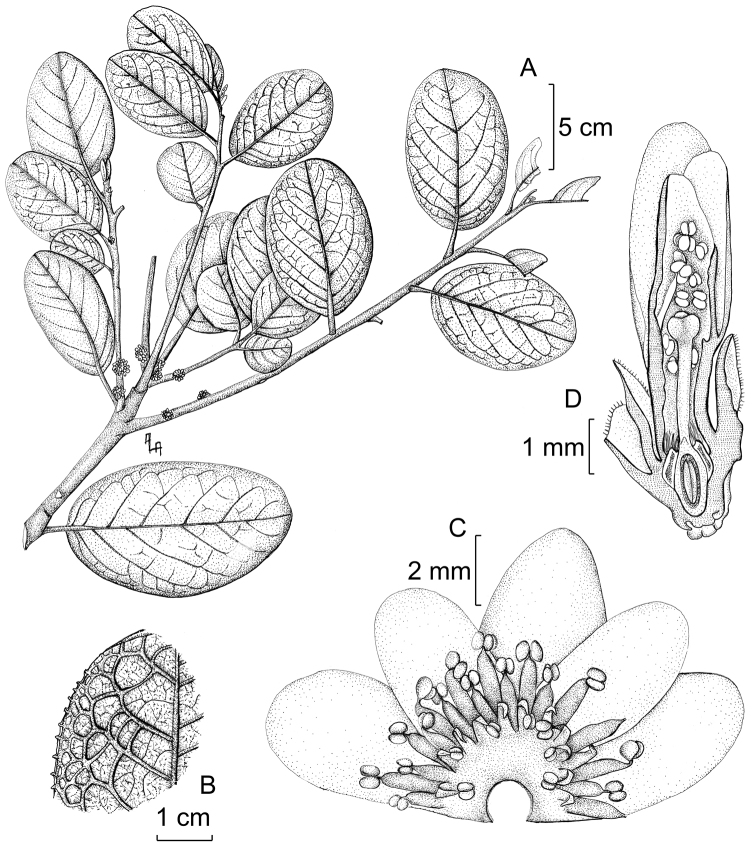
*Symplocos limonensis*. **A** Flowering branch **B** Detail of venation on leaf lower surface **C** Corolla and stamens whorls **D** Young flower in longitudinal section. (**A, B** from *Jørgensen 92876*; **C, D** from *Ulloa 487*). Illustration by A.L. Arbeláez.

#### Speciemens examined

**(Paratypes). ECUADOR. Morona-Santiago**: road Gualaceo-Limón, km 33.3, 03°02'S, 78°35'W, 3010 m, 27 Dec 1990 (fl), *P.M. Jørgensen, C. Ulloa & B. Øllgaard 92876* (AAU!, QCA!, QCNE!); Collay, Maylas, on road to Limón 03°00.36'S, 78°38.00'W, 3150 m, 14 Jun 2000 (bud), *F. Serrano, D. Minga & A. Verdugo 1459* (HA!).

#### Etymology.

The name of the species refers to the town of Limón, officially known as General Leonidas Plaza Gutiérrez. The road leading from the Andean town of Gualaceo to Limón, situated at 1400 m altitude on the east Andean slopes, has long been considered an important locality for botanical exploration where many new species have been found.

#### Distribution and conservation status.

The species is known solely from three collections made in disturbed upper Andean forests and scrub páramo on the highest point of the Gualaceo-Limón-Macas road in Morona-Santiago Province of southern Ecuador. The area of occupancy (AOO) of the species is less than 20 km^2^ and falls within the “Área de Conservación Municipal Tinajillas-Río Gualaceño,” a locally managed reserve; however, the category of protection is of lesser status than that of a National Park. Since 2000, we have searched for additional plants but could not locate any, finding only one population of *Symplocos quitensis* Brand (1901: 76), a very different species with a wide Andean distribution. The road was poorly maintained and treacherous until recently, but has now been considerably widened in the process of being paved, and consequently the natural vegetation alongside is heavily destroyed. Ongoing wood extraction for charcoal production, expansion of areas under cultivation, and mining activities for clay, gravel, and metals are threats to the natural habitats in this region. Given current knowledge, we assign a provisional IUCN conservation status of Endangered (IUCN 2014) to this species.

#### Discussion.

*Symplocos limonensis* resembles *Symplocos clethrifolia* Ståhl and *Symplocos golondrinae* Ståhl by having leaves with conspicuous venation, the veins of the lower side being prominent and verrucose and those on the upper side impressed. It differs from *Symplocos clethrifolia*, which also is restricted to southern Ecuador (but from other localities), by having larger leaves (up to 13.5 vs. 9.5 cm) with evident (i.e., not concealed) venation areoles abaxially (vs. densely ferrugineous-velutinous with matted hairs concealing areoles), sessile inflorescences (vs. pedunculate), smaller (5–6 vs. c. 8 mm long), white (vs. red to pink) corollas, and fewer stamens (40–50 vs. 60–70). From *Symplocos golondrinae* it differs by the larger size of leaves and flowers, with the leaves being coriaceous (vs. cartilaginous), long-villous abaxially (vs. sparsely strigose) and having longer (1–1.5 vs. < 1 cm) petioles, and by having a densely villous flower disk (vs. glabrous). Moreover, *Symplocos golondrinae* occurs in northwestern Ecuador, on the opposite side of the Andes.

*Symplocos quitensis* has been collected in the same area of the new species, but it is readily distinguished from both *Symplocos limonensis* and *Symplocos clethrifolia* by the densely hispid branchlets, smaller (up to 6.5 × 4 cm), membranaceous leaves, and pinkish flowers borne in short racemes.

In the most recent key to Andean *Symplocos* (Ståhl 2010a), *Symplocos limonensis* keys to *Symplocos clethrifolia* (albeit with the inflorescence pedunculate) or to *Symplocos robusta* Ståhl. The latter species is known only from Bolivia and differs from *Symplocos limonensis* in, e.g., its larger (to 14.5 × 8.5 cm), longer-petiolate (to 2 cm) leaves, more numerous flowers (up to 20) per inflorescence, and strigose corollas (vs. glabrous).

*Symplocos limonensis* has flowers with 5 to 7 petals, but being notoriously instable in many species of *Symplocos*, and often not studied, the number of petals may show to be of little taxonomic significance.

In the key to infrageneric taxa of the genus (Fritsch et al. 2008), *Symplocos limonensis* falls into the tropical American clade Symplocos subg. Symplocos sect. Symplocos having exerted, monodelphous stamens adnate to the corolla beyond its base, and the filaments flattened and constricted apically.

## Supplementary Material

XML Treatment for Symplocos limonensis

## References

[B1] Barriga P (2011) Symplocaceae. In: León-Yánez S Valencia R Pitman N Endara L Ulloa Ulloa C Navarrete H (Eds) Libro Rojo de las plantas endémicas del Ecuador, 2da. Edición Publicaciones del Herbario QCA, Pontificia Universidad Católica del Ecuador, Quito, 795−797.

[B2] Brand A (1901) IV. 242. Symplocaceae. In: Engler A von (Ed.) Das Pflanzenreich regni vegetablilis conspectus. Heft 6 W. Engelmann, Leipzig, 100 pp.

[B3] Fritsch P Kelly LM Wang Y Almeda F Kriebel R (2008) Revised infrafamilial classificaton of Symplocaceae based on phylogenetic data from DNA sequences and morphology. Taxon 57: 823–852.

[B4] Jacquin NJ von (1760) *Symplocos*. Enumeratio Systematica Plantarum. Theodorum Haak, Lugduni Batavorum [Leiden], 5 pp.

[B5] IUCN (2014) Guidelines for Using the IUCN Red List Categories and Criteria. Version 11. Prepared by the Standards and Petitions Subcommittee http://jr.iucnredlist.org/documents/RedListGuidelines.pdf [accessed: 27 Feb 2015]

[B6] Ståhl B (1991) Symplocaceae. In: Harling G Andersson L (Eds) Flora of Ecuador 43 University of Göteborg, 44 pp.

[B7] Ståhl B (1994) The genus *Symplocos* (Symplocaceae) in Bolivia. Candollea 49: 369–388.

[B8] Ståhl B (1995) New or noteworthy Andean species of the genus *Symplocos* (Symplocaceae). Candollea 50: 445–452.

[B9] Ståhl B (2010a) Four new species and records of *Symplocos* (Symplocaceae) from Peru and Bolivia, and a key to all species of *Symplocos* known to occur in Ecuador, Peru and Bolivia. Nordic Journal of Botany 28: 79–87. doi: 10.1111/j.1756-1051.2009.00445.x

[B10] Ståhl B (2010b) Additions to the knowledge of the genus *Symplocos* (Symplocaceae) in Ecuador and Peru. Novon 20: 84–94. doi: 10.3417/2008079

[B11] Thiers B (2015) Index Herbariorum: A global directory of public herbaria and associated staff. New York Botanical Garden’s Virtual Herbarium. http://sweetgum.nybg.org/ih/ [accessed: 27 Feb 2015]

